# Thoracic duct identification with indocyanine green fluorescence during minimally invasive esophagectomy with patient in prone position

**DOI:** 10.1093/dote/doaa030

**Published:** 2020-05-25

**Authors:** Massimo Vecchiato, Antonio Martino, Massimo Sponza, Alessandro Uzzau, Antonio Ziccarelli, Federico Marchesi, Roberto Petri

**Affiliations:** 1 Division of Surgery, Department of Surgery, ASUI “Santa Maria della Misericordia”, Udine, Italy; 2 Division of Angiographic Diagnostics and Interventional Radiology, Department of Diagnostics Imaging, ASUI “Santa Maria della Misericordia”, Udine, Italy, and; 3 Unit of Clinical Surgery, Department of Medicine and Surgery, University of Parma, Parma, Italy

**Keywords:** esophagectomy, prone position, thoracoscopy, thoracic duct, indocyanine green

## Abstract

Chylothorax is a serious complication of transthoracic esophagectomy. Intraoperative thoracic duct (TD) identification represents a possible tool for preventing or repairing its lesions, and it is most of the time difficult, even during high-definition thoracoscopy. The aim of the study is to demonstrate the feasibility of using near-infrared fluorescence-guided thoracoscopy to identify TD anatomy and check its intraoperative lesions during minimally invasive esophagectomy. A 0.5 mg/kg solution of indocyanine green (ICG) was injected percutaneously in the inguinal nodes of 19 patients undergoing minimally invasive esophagectomy in a prone position, before thoracoscopy. TD anatomy and potential intraoperative lesions were checked with the KARL STORZ OPAL1^®^ Technology. In all of the 19 patients where transthoracic esophagectomy was feasible, the TD was clearly identified after a mean of 52.7 minutes from injection time. The TD was cut for oncological radicality in two patients, and it was successfully ligated under the ICG guide. No postoperative chylothorax or adverse reactions from the ICG injection occurred. The TD identification with indocyanine green fluorescence during minimally invasive esophagectomy is a simple, effective, and non-time-demanding tool; it may become a standard procedure to prevent postoperative chylothorax.

## INTRODUCTION

Chylothorax is a serious postoperative complication, occurring in 2–12% of the patients submitted to esophagectomy.^1^ It delays oral intake, increases hospital stay, and negatively affects overall survival. Moreover, a thoracic duct (TD) lesion leads to a reduction of body fluids and albumin, resulting in hypovolemia^1^ and causing T-cell depletion.^2^^,^^3^ Chylothorax is also associated with pneumonia, resulting in respiratory distress,^1^ which also determines an increased risk of sepsis (24%).^1^ Conservative treatment is associated with high mortality rate (82%); however, surgical treatment exposes a fragile patient to a further thoracotomy or thoracoscopy, with possible respiratory complications, therefore affecting overall survival.^4–6^

A clear intraoperative identification of the TD is the most reasonable prophylactic measure to prevent its lesions. However, intraoperative identification of the TD course or leakage site is often difficult. The TD runs through the aortic hiatus between the azygos vein and the aorta, it then crosses the midline at the 4th to 5th thoracic vertebra and enters the left jugular–subclavian junction.^2^ Many tools have been put forward in order to better identify the TD intraoperatively.^6–8^ A preoperative anatomic reconstruction from lymphangiography and lymphoscintigraphy is difficult to transfer in the operative field. A traditional way to identify the TD and reduce iatrogenic injury is the oral administration of heavy cream or oil before surgery.^8^^,^^10^ In the last decades, the widespread diffusion of minimally invasive surgery has been supported by technological advancement in intraoperative imaging techniques. During minimally invasive esophageal surgery, the intraoperative use of indocyanine green (ICG) with near-infrared (NIR) fluorescence is an emerging technique for the evaluation of gastric conduit perfusion.^9–13^ In literature, the intraoperative use of IGC fluorescence has also been reported in open surgery, and in small reports in thoracoscopy, to detect the TD and its lesion in patients with chylothorax after esophagectomy, neck dissection^14–16^^,^^19^, or lung surgery.^17^^,^^18^ In these reports indocyanine was injected bilaterally subcutaneously in the inguinal region or into the mesentery of the small bowel.^11–13^^,^^15^

Asithate and co-workers have demonstrated a high-sensitivity intraoperative visualization of the TD anatomy during thoracotomy and video-assisted thoracoscopic surgery in animal models, with NIR fluorescence light and subcutaneous injection of ICG into the groin, the thigh (near the great saphenous vein detected by ultrasonography or palpation), and the shin (near the great saphenous vein).^13^

A clinical application of this intraoperative ICG and NIR imaging was described in two recent reports where the authors identified the TD during lateral neck dissection^14^ and thoracoscopy.^15^ In this study we report our experience in relation to the use of NIR fluorescence-guided thoracoscopy to identify the TD anatomy and check intraoperative TD’s lesions during minimally invasive esophagectomy with patients in a prone position.

## MATERIAL AND METHODS

The aim of the study is to demonstrate the feasibility to use NIR fluorescence-guided thoracoscopy to identify the TD anatomy and check for intraoperative lesions during minimally invasive esophagectomy.

Between July 2018 and January 2019, all patients with an indication for thoracoscopic esophagectomy and with the following inclusion criteria were enrolled for the study**:** patients aged 18 or over; American Society of Anesthesiologists’ (ASA) class I, II, or III; elective surgery patients; and patients’ written acceptance to be included in the study. Patients allergic to iodine, patients who cannot undergo transthoracic esophagectomy, pregnant women, and patients unable to understand the consensus were excluded.

As from 1959, ICG was approved for clinical use by the FDA. The use of NIR fluorescence imaging with ICG was approved by our Ethical Committee. Written informed consent forms were signed by all patients.

### Surgical technique

Total esophagectomy is performed using the previously described technique.^21^ After single-lumen endotracheal intubation, patients were placed in a prone position. A right pneumothorax at 8 mmHg was created with a port at the apex of the scapula. Two or three operative trocars were inserted in the right 5th and 9th intercostal spaces. The pulmonary ligament and the mediastinal pleura were divided. The azygos vein was isolated and divided at the level of its arc using a vascular stapler. Dissection of the esophagus with the periesophageal tissue and en bloc lymphadenectomy were performed caudally to the diaphragmatic hiatus and cranially to the pleural dome or to the azygos arch in case of subtotal esophagectomy, using a coagulating hook. The gastric conduit was created by a laparoscopic or laparotomic approach. After stomach mobilization, with preservation of the right vessels, the gastric conduit was constructed with multiple firings of endoscopic stapler. The celiac lymph nodes were dissected. Left cervicotomy was performed, and the upper esophagus was isolated and divided. A cervical end-to-side anastomosis was performed using a circular stapler. In Ivor Lewis procedure, esophageal dissection followed the laparoscopic time: The distal part of the esophagus was tied up with a loop, and the esophagus was then divided with an electrocautery scissor. A manual purse-string was made in the proximal esophageal stump with a Prolene 2/0, and the anvil of a circular stapler was introduced in the esophageal stump. The incision of the lower thoracic port was enlarged, and a laparoscopic wound retractor device was inserted. An intrathoracic esophagogastric anastomosis was then performed. We did not perform preventive en masse ligation, and after esophagectomy one or two chest tubes were left in the thorax.

### ICG fluorescence NIR lymphography

**Fig. 1:**
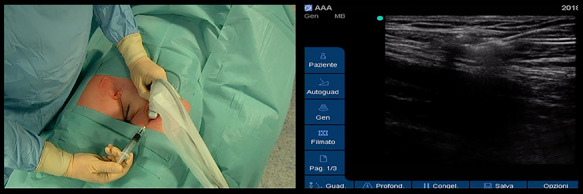
Bilateral injection of the ICG in the inguinal nodes with an ultrasound guide.

We injected 0.5 mg/kg of ICG (Diagnostic Green/indocyanine green, VERDYE 5 mg/mL) percutaneously bilaterally in the superficial inguinal nodes with an ultrasound visualization, before thoracoscopy in total esophagectomy, and after laparoscopic time in Ivor Lewis esophagectomy (Fig. 1).

The KARL STORZ OPAL1^®^ Technology for ICG-enhanced NIR was used. Using the foot pedal or left camera button, it is possible to switch from a standard mode to NIR mode during the operation and to check anatomical and potential intraoperative lesions of the TD.

TD intraoperative identification rates and chylothorax (defined as the presence of a collection of lymphatic fluid in the pleural cavity or in the thoracic drainage) were the main outcomes. The secondary outcome resulted in adverse reactions, pain, iatrogenic lesions, and the complications at injection site.

In all patients we have recorded the gender, body mass index, American Society of Anesthesia score, level of anastomosis and technique (total or subtotal esophagectomy, cervical end-to-side anastomosis, or intrathoracic esophagogastric anastomosis), operative time, adverse reactions, iatrogenic lesions, complications, or problems at the injection site.

## RESULTS

Between July 2018 and July 2019, 20 patients were enrolled for the study. Demographics and clinical data of patients are summarized in Table 1. 0.5 mg/kg of ICG diluted in 10 mL of physiological solution was injected in each patient. The ICG was injected before the thoracoscopy time. No adverse reactions or iatrogenic lesions at the injection site were observed. Mean time was about 10.5 minutes for the entire procedure (range 7–16 minutes, DS 2.6).

**Table 1:** Demographics and clinical data of patients

Patient	Age	Sex	Site of tumor	Histotype	Approach	Neoadjuvant therapy	Injection site	0.5 mg/kg	Time procedure	Time from injection and visualization (minutes)	Adverse reactions or iatrogenic lesions at the injection site	Section and ligation of the TD	Chylothorax
1	80	F	Middle	Squamous	McKeown	Chemoradiotherapy	Foot and inguinal nodes	22	15	60	No	No	No
2	70	M	Lower	Adenocarcinoma	Ivor Lewis	Chemoradiotherapy	Inguinal nodes	45	13	60	No	No	No
3	72	M	Middle	Adenocarcinoma	Transhiatal	Chemoradiotherapy	Inguinal nodes	37	10		No	No	No
4	55	M	Middle	Squamous	McKeown	Chemoradiotherapy	Inguinal nodes	32	16	80	No	Yes	No
5	53	M	Lower	Squamous	Ivor Lewis	Chemoradiotherapy	Inguinal nodes	41	10	70	No	No	No
6	51	F	Lower	Adenocarcinoma	Ivor Lewis	Chemoradiotherapy	Inguinal nodes	27	7	55	No	No	No
7	69	M	Middle	Squamous	NA	Chemoradiotherapy	Inguinal nodes	29	11	41	No	No	No
8	74	M	Lower	Squamous	McKeown	Chemoradiotherapy	Inguinal nodes	29	8	46	No	No	No
9	59	M	Lower	Adenocarcinoma	Ivor Lewis	Chemoradiotherapy	Inguinal nodes	30	10	55	No	No	No
10	67	M	Middle	Squamous	McKeown	Chemoradiotherapy	Inguinal nodes	29	15	60	No	No	No
11	67	M	Middle	Squamous	McKeown	Chemoradiotherapy	Inguinal nodes	34	9	45	No	Yes	No
12	64	M	Lower	Adenocarcinoma	Ivor Lewis	Chemoradiotherapy	Inguinal nodes	42	7	50	No	No	No
13	78	F	Superior	Squamous	McKeown	Chemoradiotherapy	Inguinal nodes	30	10	50	No	No	No
14	81	M	Middle	Squamous	McKeown	Chemoradiotherapy	Inguinal nodes	36	8	50	No	No	No
15	69	M	Lower	Adenocarcinoma	Ivor Lewis	Chemoradiotherapy	Inguinal nodes	44	8	40	No	No	No
16	54	M	Middle	Squamous	McKeown	Chemoradiotherapy	Inguinal nodes	35	12	60	No	No	No
17	75	F	Middle	Squamous	McKeown	Chemoradiotherapy	Inguinal nodes	37	9	55	No	No	No
18	78	M	Lower	Adenocarcinoma	Ivor Lewis	Chemoradiotherapy	Inguinal nodes	40	9	45	No	No	No
19	81	M	Lower	Adenocarcinoma	Ivor Lewis	Chemoradiotherapy	Inguinal nodes	38	10	45	No	No	No
20	59	M	Lower	Squamous	McKeown	Chemoradiotherapy	Inguinal nodes	22	12	45	No	Yes	No

**Fig. 2:**
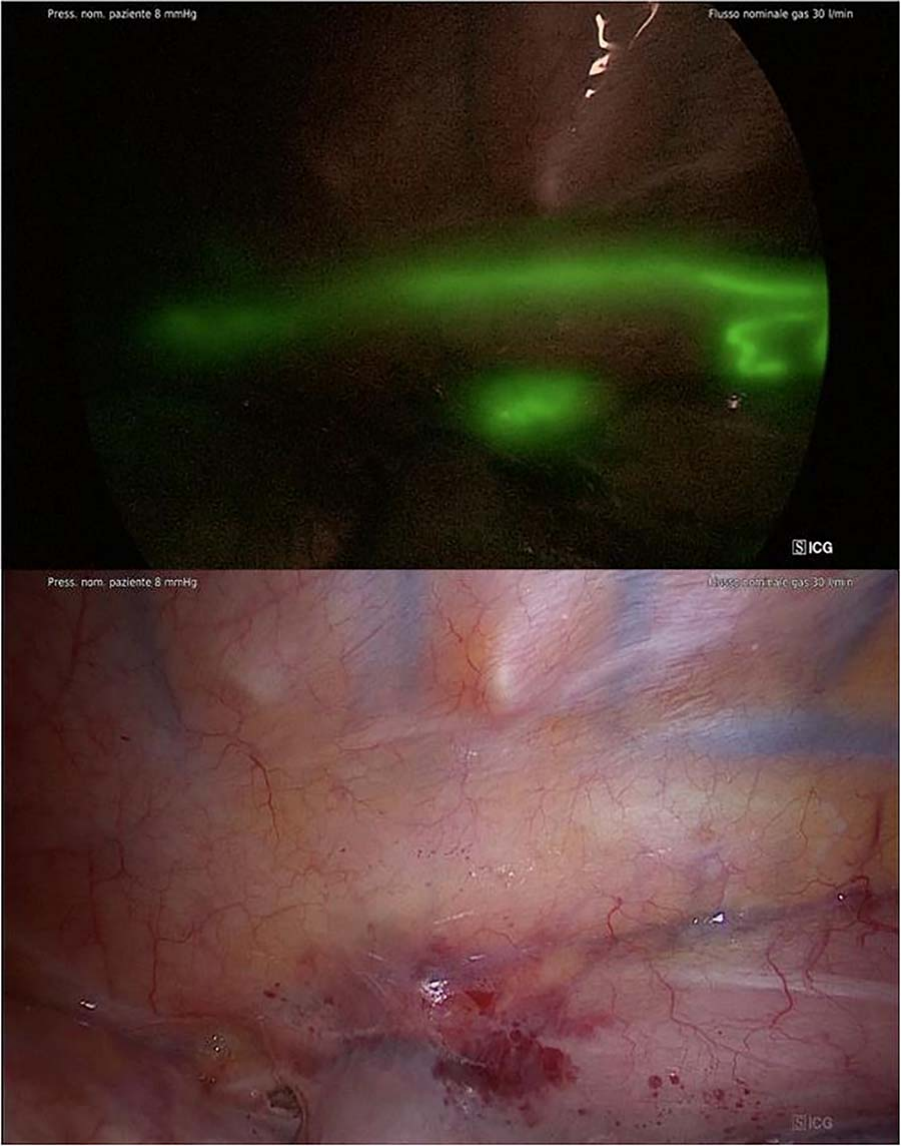
2bis Identification of TD after the injection (NIR/ICG with SPECTRA A).

**Fig. 3:**
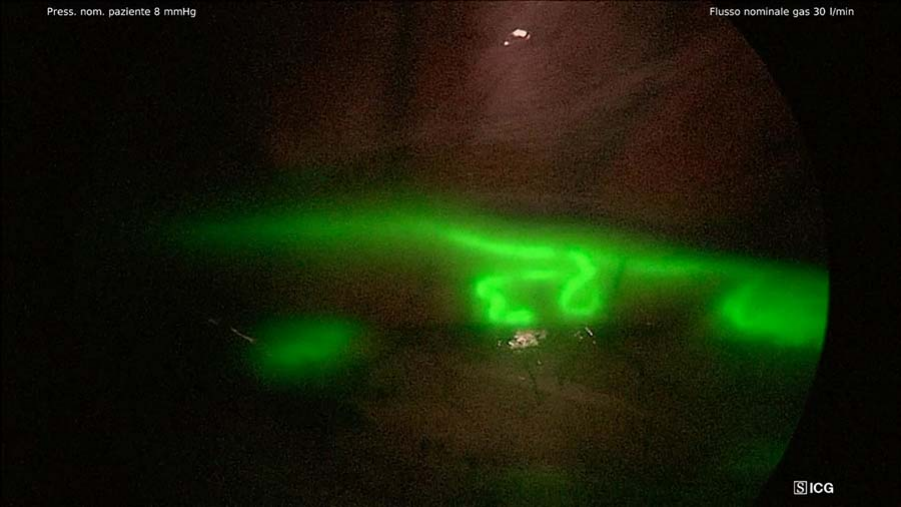
Identification of tributary and aberrant duct of TD (NIR/ICG with SPECTRA A).

One patient, after inguinal node injection of ICG, was excluded from the study because of the presence of pleural adhesion that prevented thoracoscopy and thoracotomy; a transhiatal esophagectomy was performed in this patient. In all of the 19 patients who underwent thoracoscopic esophagectomy, the TD during thoracoscopy was identified (Fig. 2-2bis) after a mean of 52.7 minutes from injection time (range 35–80 minutes, DS 10.6). The tributary of the TD and aberrant ducts were also visualized (Fig. 3). In two patients, the technique highlighted the TD retracted in a scar tissue after chemoradiation therapy, and in order to complete the esophagectomy, we had to cut it prior to ligation. Using the ICG guide, the two stumps were detected and ligated with a stitch and two clips. Effective closure was confirmed by the ICG and the NIR fluorescence. Median operative time was 258 minutes. Fluorescence was present until the end of the operation in all cases. No patient had a postoperative chylothorax. No major surgical complications occurred. The chest tubes were removed after 7 days, and the median postoperative hospital stay was 10 days.

## DISCUSSION

The technique proved to be effective for the identification of the TD (success rate 100%), with a clear anatomic visualization of the TD and the tributary and aberrant duct, as shown in Figures 2 and 3. The constant control of the anatomy along with the possibility to switch images from standard light to NIR mode makes the dissection safe and ‘comfortable’ for the surgeon. In addition, in the case where the TD was cut for oncological radicality, the ICG and NIR fluorescence helped us to easily ligate the two stumps and to check for absence of any leakage.

No adverse reactions or iatrogenic lesions at the injection site were observed. No chylothorax was recorded during postoperative stay.

Esophagectomy is the only potentially curative treatment of esophageal cancer, and even in a high-volume center, it is associated with a significant morbidity and mortality.^22^ Over the past decades, the improvement of minimally invasive approaches for esophagectomy contributed to minimize surgical trauma and complication rate.^11^^,^^21^ Since 2005 minimally invasive esophagectomy with patients in a prone position has been the standard procedure in our center, with a 2.3% mortality rate within 30 days and a morbidity rate similar to other published series.^19–23^ Chylothorax is still a rare but potentially life-threatening complication. In our experience, between July 2005 and December 2017, we had six chylothorax (3.9%) on 156 patients who underwent esophagectomy. One of those patients died due to subsequent respiratory distress syndrome with multiple organ failure.

The proper treatment procedure and timing of chylothorax is still a matter of debate.^24^

Conservative treatment (cessation of oral intake) is an option, but success rate ranges widely (3–90%), depending of the underlying cause of disease.^24^ Noninvasive or semi-invasive procedures present a variable success rate (50–100%) also depending on the etiology.^6^ Another therapeutic option is surgical treatment, but only for patients fit for surgery. Surgical therapy consists of TD ligature; pleurodesis, the placement of a permanent chest drain, or a pleuroperitoneal shunt may be performed. The success rate of these procedures ranges between 64 and 100%, but the morbidity and mortality rate can amount to 25%.^6^^,^^23^ We believe that the correct intraoperative identification of the TD anatomy and lesions is the best way to prevent chylothorax. Intraoperative TD en masse ligation, a described procedure to prevent chylothorax,^19–20^ is not a routine in our center. Indeed, the TD ligation itself is not an innocuous procedure, and it should be avoided if possibile.^24^^,^^25^^,^^28^^,^^29^ After the ligation of TD, even without chylothorax, it will make postoperative course more complicated.^26^,^28^^,^^29^ Moreover, a systematic review of Yiyan showed no evidence of reduction of postoperative chythorax with the prophylactic TD ligation.^27^

The thoracoscopic approach in a prone position allows for great exposure and visualization of the operatory field during esophageal dissection; nevertheless the TD identification is often complicated.

In fact, as previously commented, to date no standardized and effectively reliable intraoperative diagnostic tools are available for the identification of the TD.

ICG is an FDA-approved fluorescent dye with many clinical applications.^18^^,^^7^ During the last years, the development of new platforms for endoscopic imaging using ICG with NIR fluorescence promoted the implementation of a novel diagnostic approach in many fields of clinical practice. In thoracic surgery, in particular, it has been proposed for the evaluation of gastric conduit perfusion during esophagectomy,^9^^,^^11^ while there are some reports of its intraoperative use to detect TD lesions in patients with chylothorax after open esophagectomy.^4^^,^^5^^,^^12^^,^^14^^,^^17^^,^^19^ In these reports indocyanine was injected subcutaneously in the bilateral inguinal region or into the mesentery of the small bowel.^11^^,^^14^^,^^19^

Animal models have demonstrated a high-sensitivity intraoperative visualization of the TD anatomy in thoracotomy and video-assisted thoracoscopic surgery with NIR fluorescence light and subcutaneous injection of ICG^5^^,^^11^, and there are some small reports that have published the idea of using ICG fluorescence to visualize TD leakage sites.^12^^,^^15–18^

As recently reported,^17^ ultrasound-guided inguinal node injection is a safe and feasible way to obtain the TD visualization, even though more cases are needed to determine with certainty if the site of injection and the concentration used may be standardized. Moreover, in our experience, the procedure is easy to perform, not time-demanding (10 minutes), and not expensive, and the fluorescence was present during all the operative time.

Nineteen successful cases are not enough to affirm that the TD identification with ICG fluorescence during minimally invasive esophagectomy may be proposed as a standard tool to prevent chylothorax, and larger comparative studies have to be programmed, but our results are useful for standardization of the method. This technique allows a safe dissection, giving a constant anatomic feedback to the surgeon, and it can easily be reproduced. Furthermore this technique could make the preservation of TD without injury, a convincing procedure avoiding the need of prophylactic TD ligation, which may cause several postoperative problems. We need larger comparative studies, but we hope that it may be proposed as a standard tool to prevent chylothorax during esophagectomy.

## Notes

Financial disclosure: The authors have no financial disclosures to make. There were no conflicts of interest.
